# Integrated dataset of the Korean Genome and Epidemiology Study cohort with estimated air pollution data

**DOI:** 10.4178/epih.e2022071

**Published:** 2022-09-07

**Authors:** Hae Dong Woo, Dae Sub Song, Sun Ho Choi, Jae Kyung Park, Kyoungho Lee, Hui-Young Yun, Dae-Ryun Choi, Youn-Seo Koo, Hyun-Young Park

**Affiliations:** 1 Division of Population Health Research, Department of Precision Medicine, Korea National Institute of Health, Korea Disease Control and Prevention Agency, Cheongju, Korea; 2 Department of Environmental and Energy Engineering, Anyang University, Anyang, Korea; 3 Department of Precision Medicine, Korea National Institute of Health, Korea Disease Control and Prevention Agency, Cheongju, Korea

**Keywords:** Air pollution, Health, Dataset, Particulate matter

## Abstract

Public concern about the adverse health effects of air pollution has grown rapidly in Korea, and there has been increasing demand for research on ways to minimize the health effects of air pollution. Integrating large epidemiological data and air pollution exposure levels can provide a data infrastructure for studying ambient air pollution and its health effects. The Korean Genome and Epidemiology Study (KoGES), a large population-based study, has been used in many epidemiological studies of chronic diseases. Therefore, KoGES cohort data were linked to air pollution data as a national resource for air pollution studies. Air pollution data were produced using community multiscale air quality modeling with additional adjustment of monitoring data, satellite-derived aerosol optical depth, normalized difference vegetation index, and meteorological data to increase the accuracy and spatial resolution. The modeled air pollution data were linked to the KoGES cohort based on participants’ geocoded residential addresses in grids of 1 km (particulate matter) or 9 km (gaseous air pollutants and meteorological variables). As the integrated data become available to all researchers, this resource is expected to serve as a useful infrastructure for research on the health effects of air pollution.

## INTRODUCTION

Exposure to indoor and outdoor air pollution is the fourth out of 87 risk factors for premature death, according to the Global Burden of Disease study [1]. The World Health Organization (WHO) has provided new air quality guidelines based on increasing evidence that air pollution has a considerable impact on human health, even at lower concentrations [2]. East Asia, where Korea is located, has continued to maintain high levels of air pollution, which may result in a high burden of mortality and disease due to air pollution.

Public concern about the human health impacts of air pollution has increased. Hence, it is necessary to understand the adverse effects of air pollution on human health and gather scientific evidence on ways to minimize them. Large-scale epidemiological research is needed to evaluate the Korea-specific human effects of air pollution and generalize the study results. Previous large-scale epidemiological studies conducted in Korea have used secondary data from the National Statistical Office, National Health Insurance Service, and emergency room inpatient data. However, these data are difficult to control for other factors that can affect the association between air pollution and the outcomes [3-8]. Particularly, it is difficult to merge air pollution levels in the secondary data because they often do not provide detailed addresses. The Korean Genome and Epidemiology Study (KoGES) and the Korea National Health and Nutrition Examination Survey, collected by the Korea Disease Control and Prevention Agency, are excellent in terms of representativeness, scale, diversity of disease-related variables, and standardization. Building a dataset by linking air pollutant exposure levels to the collected data can provide a data infrastructure for studying ambient air pollution and its health effects.

Accurate estimates of personal exposure to pollutants are important to measure the health risks of air pollution. Data from air pollution monitoring stations based on participants’ residential addresses have been widely used because it is difficult to monitor individual exposure levels, especially in a large population group. Although the nationwide air pollution monitoring network has expanded rapidly in Korea, monitoring sites are still limited in areas other than large cities or metropolitan areas. Particulate matter (PM) acts as an indicator of air pollution, and smaller particle sizes can affect human health. Nationwide data on PM≤ 2.5 microns in diameter (PM_2.5_) from monitoring stations have been available in Korea since 2015. Therefore, the Community Multiscale Air Quality (CMAQ) model, which includes meteorological and emission modeling, can be a good modeling method because it is useful for estimating air quality levels when data from monitoring stations are not available.

Therefore, we aimed to estimate the air pollution levels nationwide and merge them with the KoGES cohort to establish a data infrastructure for the study of ambient air pollution and its health effects. Herein, we introduced a detailed procedure for estimating exposure to air pollution among the KoGES participants (and how to merge the data with the KoGES cohort) and air pollution levels.

## DATA SOURCE

### Korean Genome and Epidemiology Study cohort

The KoGES, which includes several large-scale population-based cohorts funded by the National Research Institute of Health, recruited people over 40 years of age to identify genetic and environmental factors of chronic diseases such as diabetes, hypertension, obesity, hyperlipidemia, metabolic syndrome, and cardiovascular disease. Baseline recruitment for the KoGES was conducted between 2001 and 2013 [9]. The KoGES consists of population-based and gene-environment model studies. In population-based studies, the KoGES Ansan and Ansung study, the KoGES Cardiovascular Disease Association Study (CAVAS), and the KoGES Health Examinees (HEXA) study were used as epidemiological data to combine with air pollution exposure levels.

For the KoGES Ansan and Ansung study, recruitment was carried out in 2 different regions, representative of industrialized communities (Ansan) and rural areas (Ansung). The basic survey was started between 2001 and 2002, and follow-up with participants continued every 2 years. For the KoGES CAVAS, recruitment was carried out from multiple rural communities. The study completed the fourth follow-up in 2016 after the baseline survey from 2005 to 2011. A total of 211,569 participants provided consent to participate in the cohort study as of May 2022, after excluding those who withdrew from the study. The KoGES cohort data included general characteristics, medical history, smoking and drinking status, diet questionnaire, and clinical measurements, such as vital signs and blood and urine tests. The cohort data continued to produce new variables using collected specimens. A more detailed description of the cohort has been published elsewhere [9], including a comprehensive list of data collection methods in the supplementary file. Genetic information on several platforms [10,11] that can be integrated with various KoGES data is also available. The up-to-date recruitment status of the KoGES population-based studies is presented in Table 1, and the number of participants in the 17 provinces of Korea at baseline cohort is shown in Supplemental Material 1.

## MEASURES

### Geocoding of the cohort participants

**Figure 1.**
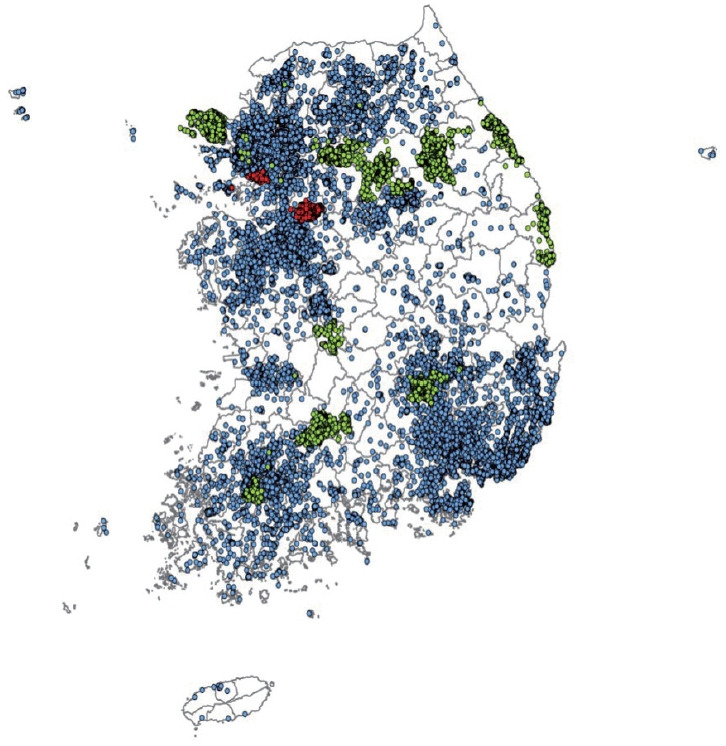
Map of the residential addresses of the participants from 3 cohort studies: KoGES Ansan and Ansung study (red), KoGES CAVAS (green), and KoGES HEXA (blue). KoGES, Korean Genome and Epidemiology Study; CAVAS, Cardiovascular Disease Association Study; HEXA, Health Examinees Study.

The cohort participants’ address information for each follow-up visit was converted into latitude and longitude coordinates using the GeoService-Xr geocoding software (Geoservice, Seoul, Korea). If there was no address in a specific follow-up survey, the address from the previous survey was used. When multiple addresses existed in the same survey, the last address entered was selected. When a detailed address was unavailable or out of date, the address was replaced with a nearby public (administrative) institution. Finally, a total of 353,646 addresses of the cohort participants from baseline to follow-up surveys of 3 population-based studies were geocoded according to the above principle, excluding 13,975 addresses with incorrect information. The geocoded addresses are presented in Figure 1.

### Geographic estimation of air pollution

Air pollution concentration was calculated in 9-km grid units using the CMAQ model, which uses meteorological information and emission rates. Thereafter, the data were assimilated using measured data from the monitoring station. For PM data, satellite-derived aerosol optical depth (AOD) was applied to increase the spatial resolution to a 1-km grid unit. Additionally, multiple linear regression was applied to PM and ozone (O_3_).

The CMAQ model includes a meteorological model, emission model, and chemical transport model. Three-dimensional meteorological data, such as the hourly wind, temperature, and humidity fields, were generated in each grid using Weather Research Forecast version 3.6.1. The generated values were used as input data for the emission and chemical transport models and as final meteorological variables.

Sparse matrix operator kernel emissions were used to process the emission data for the input of the air quality model. The sources for the emission model were the Clean Air Policy Support System for Korean emissions, the Multi-Resolution Emission Inventory for China, and the Regional Emission Inventory in Asia for emissions from neighboring regions. The chemical transport model integrates the output from the meteorological and emission models to calculate the concentration of air pollutants.

Data assimilation, a method of combining the estimated value with the observed data, was applied by assigning a weight to each observation within the radius of influence. Data were generated in 9-km grid units. However, the PM was calculated in 1-km grid units after applying AOD observations from the National Aeronautics and Space Administration Terra and Aqua satellites. Finally, PM and O_3_ were adjusted using the normalized difference vegetation index and meteorological data using multiple linear regression. More details on the modeling method can be found in a previous study [12]. The air pollution data by units of small-scale administrative divisions in Korea (*dong* [neighborhood] or *si-gun-gu* [city-county-district] levels) were also calculated by summing the concentration of grid cells by weighting the area covering the administrative division. Administrative division data can be used to link epidemiological data that only include administrative division information without the geocoded addresses of participants. O_3_ is generally calculated as the daily maximum 8-hour mean concentration; however, the 24-hour mean O_3_ concentration was calculated in this study because of the characteristics of the model used.

The estimated air pollution data were compared with the measured data from monitoring stations using the average values of certain periods (day, week, and annual) in each grid (Table 2). The concentration at the modeling grid point where the measuring station was located was extracted and evaluated using the following statistical indices: correlation coefficient, the square of the correlation coefficient, index of agreement, and root mean square error. Although the degree of consistency between the measurement data and the modeling data was quite high, there were some differences depending on the type of air pollutants and the average period, which should be considered when interpreting the data.

The estimated air pollution levels in Korea region from 2005 to 2017 are presented in Table 3. The mean concentrations of 6 air pollutants in Korea region were calculated and compared to the air quality guideline levels of Korea and the WHO. The air quality guideline annual mean levels of PM_2.5_ and ≤ 10 microns in diameter (PM_10_) are 15 μg/m^3^ and 50 μg/m^3^, according to the enforcement decree of the framework act on environmental policy in Korea, and 5 μg/m^3^ and 15 μg/m^3^ according to the WHO [2], respectively. The concentrations of PMs have been decreasing over the years, but they are still higher than both the Korean and WHO guideline levels. The levels of gaseous air pollutants were generally lower than recommended by the guidelines, except for nitrogen dioxide (NO_2_), which was at the WHO levels.

### Merging the estimated air pollution data to the Korean Genome and Epidemiology Study cohort

The geocoded addresses of the KoGES participants were spatially matched to the air pollution data from the 1-km or 9-km grid units using the ArcGIS program (ESRI Inc., Redland. CA, USA). Various exposure periods were calculated, including the data on the day of the cohort survey. The exposure periods covered long-term and short-term exposure to air pollutants, according to previously published studies. Meteorological data were also included in the dataset because meteorological variables such as temperature and humidity are highly correlated with air pollution and have their own effects on health.

The air pollution exposure dataset consisted of 210 variables, including each exposure period (35 types) for 6 air pollutants (PM_10_, PM_2.5_, NO_2_, sulfur dioxide [SO_2_], O_3_, and carbon monoxide [CO]). The exposure period was the day of the survey (lag0), the day before the survey to 14 days (lag1 to lag14), the moving average of 1 week, 1 month, 3 months, 6 months, 1 year, 2 years, and 3 years before the survey, and the average for each calendar year from 2005 to 2017. Meteorological data such as relative humidity, wind speed, precipitation, cloudiness, insolation, and surface pressure were included in the same exposure periods as the air pollution data, except for wind direction and temperature. Average exposure was not included for wind direction, whereas the highest and lowest temperatures from the day to 14 days before the survey were included. The variables included in the dataset and their descriptions are presented in Supplemental Material 2. These datasets were separately established for each baseline and follow-up survey.

If the address of a cohort participant changed during the follow-up survey, the average exposure concentration was calculated based on the assumption that the participant moved at the midpoint between the previous and current surveys. For example, if a participant’s address changed in the following survey and the interval from the previous survey is 2 years, the participant was considered to have moved 1 year ago.

### Air pollution exposure levels of the cohort participants

**Figure 2.**
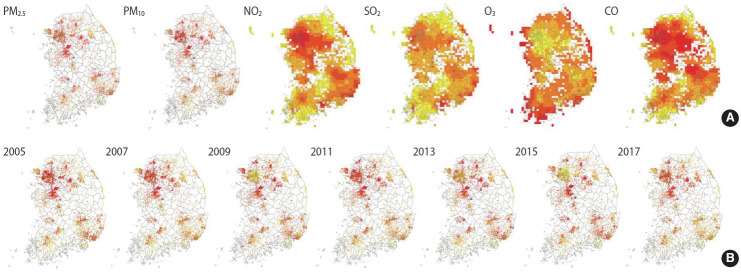
Relative air pollution levels among the KoGES cohort participants. The exposure levels were calculated using the mean exposure levels of (A) 6 pollutants during 13 years and (B) PM_2.5_ in each year (presented in every other year). The exposure levels were divided into quintiles. The highest quantile is indicated in red, the lowest quantile in yellow, and “no participant within the grid cell” in white (no color). The mean exposure levels were calculated using all data in each grid regardless of the survey date. Thus, a 1-km grid was used for PM_2.5_ and PM_10_, while a 9-km grid was used for NO_2_, SO_2_, O_3_, and CO. KoGES, The Korean Genome and Epidemiology Study; PM_2.5_, particulate matter ≤2.5 microns in diameter; PM_10_, particulate matter ≤10 microns in diameter; NO_2_, nitrogen dioxide; SO_2_, sulfur dioxide; O_3_, ozone; CO, carbon monoxide.

A total of 197,788 cohort participants (10,018 from the KoGES Ansan and Ansung study; 28,203 from the KoGES CAVAS; 159,567 from the KoGES HEXA) were merged with the air pollution data (2005-2017), excluding data from 211,569 KoGES cohort participants with unknown addresses. The mean baseline exposure levels of the cohort participants to the 6 air pollutants on the day of the survey are shown in Table 4. Detailed information on the exposure levels in the baseline survey is shown in Supplemental Materials 3 and 4. The exposure levels were higher in the spring and winter, except for O_3_. In addition, PM levels were higher in KoGES Ansan and Ansung, whereas NO_2_ levels were lower in KoGES CAVAS, which is a rural-based cohort (Supplementary Materials 5-10). To compare the relative pollutant levels in Korea, the mean exposure levels of 13 years in each grid, regardless of the survey date, were divided into quintiles. The highest quantile was indicated in red, the lowest in yellow, and “no participant within the grid cell” in white (Figure 2A). The exposure levels of PMs were higher in metropolitan and surrounding areas. The exposure level of NO_2_, which is related to traffic, was high in urban areas. Areas around harbor regions had high SO_2_ levels, whereas coastal areas had high O_3_ exposure levels. The average level of PM2.5 in each year was presented to determine the spatial distribution and trends of relative air pollution exposure levels of the KoGES participants throughout the 13 years (Figure 2B).

### Ethics statement

The study protocol was approved by the Institutional Review Board of the Korea Disease Control and Prevention Agency (No. 2019-05-04-2C-A). Written informed consent for participation in the KoGES cohort was obtained from all study participants and was confirmed by the Institutional Review Board.

## STRENGTHS AND WEAKNESSES

A high concentration of air pollution over a short period can have an immediate effect on human health. However, long-term exposure to air pollution at relatively low concentrations may have a delayed effect. Thus, research on the long-term effects on human health is important for the general population. Short-term effects of air pollution on human health can be analyzed using summary results from public data. However, research on the long-term exposure to air pollution requires epidemiological data that includes various health-related information as covariates or outcomes of the study. Thus, an integrated dataset of the KoGES and air pollution has been established. It has the following strengths: First, the KoGES is continually followed-up. In particular, the KoGES Ansan and Ansung study has been followed-up for over 20 years at 2-year intervals, which makes it possible to see the trends and cumulative effects of air pollution on health. During the 9 follow-ups, 91.2% of the baseline cohort participants visited again at least once, while 39.2% (3,995 out of 10,030) completed all the follow-up visits. Furthermore, 59.3% and 40.5% of KoGES CAVAS and KoGES HEXA cohort participants completed at least 1 visit after the baseline, respectively. Second, the KoGES includes large population-based cohorts and covers a wide range of Korea, including urban, rural, and industrialized areas, as presented in Figure 1. However, a systemic sampling procedure was not applied to represent the Korean population. Third, the KoGES cohort data contain an extensive range of health-related and disease-related information, including lifestyle factors, anthropometric and clinical measurements, medical history, and genetic information, which makes it possible to control for sources of residual confounding other than the risk factors and outcomes of interest. Fourth, the KoGES contains various clinical variables, including inflammation and oxidative stress markers, which are closely related to air pollution. In addition, Korean chip data, which are suitable for genome research in Korea, are also available, making it possible to conduct gene-environmental interaction studies. Fifth, the KoGES data are linked to health outcome data from the National Statistical Office, the National Health Insurance Service, and the National Cancer Center, making it possible to study the effects of air pollution on the incidence and mortality of various diseases. The cause of death data from the national statistics office can only be accessed by the researchers presently. Finally, the estimated air pollution and meteorological data estimated by the grid and administrative division unit are also available. They can be merged with other epidemiological data for various research purposes.

However, caution should be exercised when using an integrated database, owing to several limitations. First, PM_2.5_, which is known to have more adverse effects than PM_10_, may not have been fully adjusted for by the data assimilation method during 2005-2014 due to the lack of monitoring data. However, current air pollution estimations use the CMAQ model, which does not draw upon monitoring data, and additional adjustments were applied with satellite-driven AOD, normalized difference vegetation index, and meteorological variables to assess the precise concentrations of air pollution. Second, the degree of consistency between the measurement data and the modeling data was relatively low for the annual exposure of PM2.5, which may be related to the lack of monitoring data compared to other pollutants. This should be considered when interpreting the long-term exposure data. Third, personal exposure levels were estimated based on the participants’ residential addresses without considering personal space-time activity patterns. Lastly, the exposure levels of gaseous-phase air pollution are estimated in 9-km grid units, according to which all participants within each 9-km grid unit would have the same pollution levels, which may cause area-level confounding.

Even within the same area, the air pollution exposure level can vary according to the proximity of traffic routes. In particular, metropolitan areas are highly populated, and residential areas are close to traffic routes. Therefore, we plan to estimate air pollution levels with a smaller spatial resolution, using 100-m grid units, and to establish an integrated dataset with recent data. However, a more accurate method for assessing individual air pollution levels is to use personal monitoring sensors. Air pollution monitoring sensors have been developed to assess personal exposure levels. However, they should be upgraded to be smaller, lighter, and convenient for use with precise assessments, which can be utilized in a large population in the future.

## DATA ACCESSIBILITY

Researchers who wish to use the integrated dataset or the estimated air pollution and meteorological data can access the KoGES epidemiological data online sharing system (http://www.kdca.go.kr/research/KoGES/data sharing, https://nih.go.kr/ko/main/contents.do?menuNo=300566) or the National Biobank of Korea (http://www.nih.go.kr/biobank/assess&sharing). A guidebook for using the integrated dataset is also available in the online sharing system.

## Figures and Tables

**Table 1.** Up-to-date recruitment status of KoGES population-based studies (n=211,569)^1^

Status	KoGES Ansan and Ansung^2^	KoGES CAVAS^2^	KoGES HEXA^2^
Baseline	10,030 (2001-2002)	28,337 (2004-2013)	173,202 (2004-2013)
Follow-up			
1st	8,603 (2003-2004)	12,463 (2007-2014)	65,611 (2012-2016)
2nd	7,515 (2005-2006)	11,399 (2008-2016)	
3rd	6,688 (2007-2008)	6,423 (2011-2016)	
4th	6,665 (2009-2010)	1,449 (2014-2016)	
5th	6,238 (2011-2012)		
6th	5,906 (2013-2014)		
7th	6,318 (2015-2016)		
8th	6,157 (2017-2018)		
9th	5,854 (2019-2020)		
10th	Ongoing (2021-2022)		

KoGES, Korean Genome and Epidemiology Study; CAVAS, Cardiovascular Disease Association Study; HEXA, Health Examinees Study.

1 As of May 2022, after excluding those who withdrew from the study.

2 Number (survey year).

**Table 2.** Comparison between estimated and monitoring site data

Variables	Day				Week				Year			
	R	R^2^	RMSE	IOA	R	R^2^	RMSE	IOA	R	R^2^	RMSE	IOA
PM_10_ (μg/m^3^)	0.88	0.78	15.16	0.92	0.88	0.77	10.35	0.91	0.64	0.42	6.56	0.75
PM_2.5_ (μg/m^3^)	0.81	0.66	8.31	0.88	0.78	0.60	6.26	0.85	0.50	0.25	4.09	0.65
NO_2_ (ppm)	0.86	0.74	0.0069	0.93	0.86	0.73	0.0058	0.92	0.86	0.74	0.0044	0.92
SO_2_ (ppm)	0.68	0.46	0.0030	0.81	0.69	0.47	0.0025	0.81	0.66	0.43	0.0020	0.77
CO (ppm)	0.83	0.69	0.16	0.89	0.82	0.68	0.14	0.87	0.75	0.57	0.10	0.79
O_3_ (ppm)	0.81	0.66	0.0072	0.89	0.86	0.74	0.0052	0.92	0.67	0.47	0.0032	0.77

RMSE, root mean square error; IOA, index of agreement; PM_10_, particulate matter ≤10 microns in diameter; PM_2.5_, particulate matter ≤2.5 microns in diameter; NO_2_, nitrogen dioxide; SO_2_, sulfur dioxide; CO, carbon monoxide; O_3_, ozone.

**Table 3.** Estimated mean concentrations of air pollutants in Korea

Year	PM_2.5_ (μg/m^3^)	PM_10_ (μg/m^3^)	NO_2_ (ppm)	SO_2_ (ppm)	O_3_ (ppm)	CO (ppm)
2005	26.4± 12.4	53.1±22.0	0.020±0.013	0.005±0.003	0.022±0.008	0.512±0.260
2006	27.2±14.3	54.7±34.1	0.021±0.013	0.005±0.003	0.023±0.011	0.560±0.270
2007	26.4±13.3	54.2±31.8	0.021±0.014	0.006±0.004	0.023±0.010	0.526±0.281
2008	24.7±12.5	52.6±24.7	0.021±0.014	0.005±0.003	0.024±0.009	0.497±0.242
2009	24.8±12.0	51.1±24.2	0.020±0.013	0.005±0.003	0.027±0.012	0.494±0.241
2010	24.5±12.5	48.4±22.9	0.020±0.013	0.004±0.003	0.023±0.009	0.442±0.223
2011	25.1±11.8	49.3±24.8	0.020±0.013	0.004±0.003	0.024±0.010	0.415±0.206
2012	25.5±12.4	44.8±18.4	0.020±0.013	0.005±0.003	0.026±0.010	0.445±0.203
2013	25.2±12.0	49.2±21.5	0.020±0.013	0.005±0.003	0.028±0.012	0.476±0.220
2014	24.4±12.5	46.3±20.6	0.020±0.013	0.005±0.003	0.028±0.011	0.441±0.203
2015	25.0±13.0	47.8±24.2	0.019±0.012	0.004±0.003	0.028±0.012	0.463±0.192
2016	25.1±10.4	44.5±17.0	0.017±0.012	0.004±0.002	0.028±0.011	0.427±0.148
2017	23.3±10.7	43.6±17.7	0.019±0.012	0.004±0.002	0.030±0.011	0.417±0.160
All	25.2±12.4	49.2±24.1	0.020 ±0.013	0.005±0.003	0.026±0.011	0.470±0.227

Values are presented as the mean±standard deviation; Means were calculated based on the data by the administrative division to include only Korean land areas, without including sea areas. PM_2.5_, particulate matter ≤2.5 microns in diameter; PM_10_, particulate matter ≤10 microns in diameter; NO_2_, nitrogen dioxide; SO_2_, sulfur dioxide; O_3_, ozone; CO, carbon monoxide.

**Table 4.** The baseline air pollution exposure levels on the date of the survey (lag0) and 1 year moving average among the cohort participants

Variables	KoGES Ansan and Ansung^1^	KoGES CAVAS	KoGES HEXA
The date of the survey (lag0), n	7,514	28,201	159,246
PM_2.5_ (μg/m^3^)	32.52±15.45	25.49±13.18	25.86±12.93
PM_10_ (μg/m^3^)	59.60±25.71	48.72±23.08	50.68±23.33
NO_2_ (ppm)	0.0227±0.0109	0.0155±0.0094	0.0248±0.0132
SO_2_ (ppm)	0.0047±0.0022	0.0047±0.0033	0.0052±0.0033
O_3_ (ppm)	0.0218±0.0085	0.0214±0.0093	0.0223±0.0098
CO (ppm)	0.5006±0.1873	0.5527±0.3199	0.4890±0.2243
1-yr moving average, n	3,910	26,915	143,507
PM_2.5_ (μg/m^3^)	33.27±1.59	26.26±3.30	26.29±3.32
PM_10_ (μg/m^3^)	66.90±2.29	53.18±6.38	53.33±6.66
NO_2_ (ppm)	0.0254±0.0033	0.0149±0.0052	0.0251±0.0085
SO_2_ (ppm)	0.0055±0.0007	0.0044±0.0013	0.0055±0.0019
O_3_ (ppm)	0.0188±0.0007	0.0247±0.0034	0.0228±0.0033
CO (ppm)	0.6022±0.0206	0.4965±0.1046	0.5130±0.0993

Values are presented as mean±standard deviation. KoGES, Korean Genome and Epidemiology Study; CAVAS, Cardiovascular Disease Association Study; HEXA, Health Examinees Study; PM_2.5_, particulate matter ≤2.5 microns in diameter; PM_10_, particulate matter ≤10 microns in diameter; NO_2_, nitrogen dioxide; SO_2_, sulfur dioxide; O_3_, ozone; CO, carbon monoxide.

1 The second follow-up was considered the baseline for the KoGES Ansan-Ansung study because air pollution levels have been estimated since 2005.

## SUPPLEMENTARY MATERIALS

Supplementary Material 1. The number of participants by 17 provinces of Korea at baseline cohort

Supplementary Material 2. Variable list of the KoGES-air pollution dataset

Supplementary Material 3. Mean exposure levels of PMs among cohort participants in baseline survey

Supplementary Material 4. Mean exposure levels of gaseous air pollutants among cohort participants in baseline survey

Supplementary Material 5. PM_2.5_ exposure levels of the KoGES baseline participants by season and year. KoGES Ansan and Ansung study (2^nd^ follow-up, gray), KoGES CAVAS (green), KoGES HEXA (blue). Sp: Spring, S: Summer, F: Fall, W: Winter

Supplementary Material 6. PM_10_ exposure levels of the KoGES baseline participants by season and year. KoGES Ansan and Ansung study (2^nd^ follow-up, gray), KoGES CAVAS (green), KoGES HEXA (blue). Sp: Spring, S: Summer, F: Fall, W: Winter

Supplementary Material 7. NO_2_ exposure levels of the KoGES baseline participants by season and year. KoGES Ansan and Ansung study (2^nd^ follow-up, gray), KoGES CAVAS (green), KoGES HEXA (blue). Sp: Spring, S: Summer, F: Fall, W: Winter

Supplementary Material 8. SO_2_ exposure levels of the KoGES baseline participants by season and year. KoGES Ansan and Ansung study (2^nd^ follow-up, gray), KoGES CAVAS (green), KoGES HEXA (blue). Sp: Spring, S: Summer, F: Fall, W: Winter

Supplementary Material 9. O_3_ exposure levels of the KoGES baseline participants by season and year. KoGES Ansan and Ansung study (2^nd^ follow-up, gray), KoGES CAVAS (green), KoGES HEXA (blue). Sp: Spring, S: Summer, F: Fall, W: Winter

Supplementary Material 10. CO exposure levels of the KoGES baseline participants by season and year. KoGES Ansan and Ansung study (2^nd^ follow-up, gray), KoGES CAVAS (green), KoGES HEXA (blue). Sp: Spring, S: Summer, F: Fall, W: Winter
