# Supplementary material for: Integrated dataset of the Korean Genome and Epidemiology Study cohort with estimated air pollution data
Source: Epidemiol Health. 2022 Sep 7;44:e2022071. doi: 10.4178/epih.e2022071 (PMC9849844; doi:10.4178/epih.e2022071)
Supplement: Supplementary Material 1. — The number of participants by 17 provinces of Korea at baseline cohort [file epih-44-e2022071-suppl1.docx]

Supplementary Material 1. The number of participants by 17 provinces of Korea at baseline cohort

|  | KoGES Ansan and Ansung | KoGES CAVAS | KoGES HEXA |
| --- | --- | --- | --- |
|  | 10,030 (2001~2002) | 28,337 (2004~2013) | 173,202 (2004~2013) |
| Seoul |  |  | 35,028 |
| Busan |  |  | 24,917 |
| Incheon |  | 4,164 | 12,109 |
| Daegu |  | 6 | 8,990 |
| Daejeon |  |  | 1,330 |
| Gwangju |  | 1 | 6,531 |
| Ulsan |  |  | 5,873 |
| Sejong |  |  | 201 |
| Gyeonggi-do | 10,018 | 3,554 | 20,525 |
| Gangwon-do |  | 8,090 | 8,685 |
| Chungcheongnam-do |  | 1,998 | 10,986 |
| Chungcheongbuk-do |  |  | 2,714 |
| Jeollanam-do |  | 1,915 | 3,123 |
| Jeollabuk-do |  | 3,949 | 1,558 |
| Gyeongsangnam-do |  |  | 13,260 |
| Gyeongsangbuk-do |  | 4,524 | 3,706 |
| Jeju-do |  |  | 11 |
| N/A | 12 | 136 | 13,655 |

CAVAS: Cardiovascular Disease Association Study; HEXA, Health Examinee Study. N/A: Participants with no
