# Supplementary material for: Integrated dataset of the Korean Genome and Epidemiology Study cohort with estimated air pollution data
Source: Epidemiol Health. 2022 Sep 7;44:e2022071. doi: 10.4178/epih.e2022071 (PMC9849844; doi:10.4178/epih.e2022071)
Supplement: Supplementary Material 2. — Variable list of the KoGES-air pollution dataset [file epih-44-e2022071-suppl2.docx]

Supplementary Material 2. Variable list of the KoGES-air pollution dataset

| Variable | Description of the variable |
| --- | --- |
| P_ID | Participant’s ID |
| SIDONM | Administrative division (si-do) |
| SGGNM | Administrative division (si-gun-gu) |
| *Air pollution variables* |  |
| PM10_LAG_0 | PM10 (㎍/㎥), the day of the survey |
| PM10_LAG_1 | PM10 (㎍/㎥), 1 day before the survey |
| PM10_LAG_2 | PM10 (㎍/㎥), 2 days before the survey |
| PM10_LAG_3 | PM10 (㎍/㎥), 3 days before the survey |
| PM10_LAG_4 | PM10 (㎍/㎥), 4 days before the survey |
| PM10_LAG_5 | PM10 (㎍/㎥), 5 days before the survey |
| PM10_LAG_6 | PM10 (㎍/㎥), 6 days before the survey |
| PM10_LAG_7 | PM10 (㎍/㎥), 7 days before the survey |
| PM10_LAG_8 | PM10 (㎍/㎥), 8 days before the survey |
| PM10_LAG_9 | PM10 (㎍/㎥), 9 days before the survey |
| PM10_LAG_10 | PM10 (㎍/㎥), 10 days before the survey |
| PM10_LAG_11 | PM10 (㎍/㎥), 11 days before the survey |
| PM10_LAG_12 | PM10 (㎍/㎥), 12 days before the survey |
| PM10_LAG_13 | PM10 (㎍/㎥), 13 days before the survey |
| PM10_LAG_14 | PM10 (㎍/㎥), 14 days before the survey |
| PM10_AVG_W1 | PM10 (㎍/㎥), moving average of 1 week (Lag0~Lag6) |
| PM10_AVG_M1 | PM10 (㎍/㎥), moving average of 1 month (Lag0~Lag29) |
| PM10_AVG_M3 | PM10 (㎍/㎥), moving average of 3 months (Lag0~Lag89) |
| PM10_AVG_M6 | PM10 (㎍/㎥), moving average of 6 months (Lag0~Lag179) |
| PM10_AVG_Y1 | PM10 (㎍/㎥), moving average of 1 year (Lag0~Lag364) |
| PM10_AVG_Y2 | PM10 (㎍/㎥), moving average of 2 years (Lag0~Lag729) |
| PM10_AVG_Y3 | PM10 (㎍/㎥), moving average of 3 years (Lag0~Lag1094) |
| PM10_AVG_Y2005 | PM10 (㎍/㎥), year mean of 2005 |
| PM10_AVG_Y2006 | PM10 (㎍/㎥), year mean of 2006 |
| PM10_AVG_Y2007 | PM10 (㎍/㎥), year mean of 2007 |
| PM10_AVG_Y2008 | PM10 (㎍/㎥), year mean of 2008 |
| PM10_AVG_Y2009 | PM10 (㎍/㎥), year mean of 2009 |
| PM10_AVG_Y2010 | PM10 (㎍/㎥), year mean of 2010 |
| PM10_AVG_Y2011 | PM10 (㎍/㎥), year mean of 2011 |
| PM10_AVG_Y2012 | PM10 (㎍/㎥), year mean of 2012 |
| PM10_AVG_Y2013 | PM10 (㎍/㎥), year mean of 2013 |
| PM10_AVG_Y2014 | PM10 (㎍/㎥), year mean of 2014 |
| PM10_AVG_Y2015 | PM10 (㎍/㎥), year mean of 2015 |
| PM10_AVG_Y2016 | PM10 (㎍/㎥), year mean of 2016 |
| PM10_AVG_Y2017 | PM10 (㎍/㎥), year mean of 2017 |
| PM25_LAG_0 | PM2.5 (㎍/㎥), the day of the survey |
| PM25_LAG_1 | PM2.5 (㎍/㎥), 1 day before the survey |
| PM25_LAG_2 | PM2.5 (㎍/㎥), 2 days before the survey |
| PM25_LAG_3 | PM2.5 (㎍/㎥), 3 days before the survey |
| PM25_LAG_4 | PM2.5 (㎍/㎥), 4 days before the survey |
| PM25_LAG_5 | PM2.5 (㎍/㎥), 5 days before the survey |
| PM25_LAG_6 | PM2.5 (㎍/㎥), 6 days before the survey |
| PM25_LAG_7 | PM2.5 (㎍/㎥), 7 days before the survey |
| PM25_LAG_8 | PM2.5 (㎍/㎥), 8 days before the survey |
| PM25_LAG_9 | PM2.5 (㎍/㎥), 9 days before the survey |
| PM25_LAG_10 | PM2.5 (㎍/㎥), 10 days before the survey |
| PM25_LAG_11 | PM2.5 (㎍/㎥), 11 days before the survey |
| PM25_LAG_12 | PM2.5 (㎍/㎥), 12 days before the survey |
| PM25_LAG_13 | PM2.5 (㎍/㎥), 13 days before the survey |
| PM25_LAG_14 | PM2.5 (㎍/㎥), 14 days before the survey |
| PM25_AVG_W1 | PM2.5 (㎍/㎥), moving average of 1 week (Lag0~Lag6) |
| PM25_AVG_M1 | PM2.5 (㎍/㎥), moving average of 1 month (Lag0~Lag29) |
| PM25_AVG_M3 | PM2.5 (㎍/㎥), moving average of 3 months (Lag0~Lag89) |
| PM25_AVG_M6 | PM2.5 (㎍/㎥), moving average of 6 months (Lag0~Lag179) |
| PM25_AVG_Y1 | PM2.5 (㎍/㎥), moving average of 1 year (Lag0~Lag364) |
| PM25_AVG_Y2 | PM2.5 (㎍/㎥), moving average of 2 years (Lag0~Lag729) |
| PM25_AVG_Y3 | PM2.5 (㎍/㎥), moving average of 3 years (Lag0~Lag1094) |
| PM25_AVG_Y2005 | PM2.5 (㎍/㎥), year mean of 2005 |
| PM25_AVG_Y2006 | PM2.5 (㎍/㎥), year mean of 2006 |
| PM25_AVG_Y2007 | PM2.5 (㎍/㎥), year mean of 2007 |
| PM25_AVG_Y2008 | PM2.5 (㎍/㎥), year mean of 2008 |
| PM25_AVG_Y2009 | PM2.5 (㎍/㎥), year mean of 2009 |
| PM25_AVG_Y2010 | PM2.5 (㎍/㎥), year mean of 2010 |
| PM25_AVG_Y2011 | PM2.5 (㎍/㎥), year mean of 2011 |
| PM25_AVG_Y2012 | PM2.5 (㎍/㎥), year mean of 2012 |
| PM25_AVG_Y2013 | PM2.5 (㎍/㎥), year mean of 2013 |
| PM25_AVG_Y2014 | PM2.5 (㎍/㎥), year mean of 2014 |
| PM25_AVG_Y2015 | PM2.5 (㎍/㎥), year mean of 2015 |
| PM25_AVG_Y2016 | PM2.5 (㎍/㎥), year mean of 2016 |
| PM25_AVG_Y2017 | PM2.5 (㎍/㎥), year mean of 2017 |
| SO2_LAG_0 | SO2 (ppm), the day of the survey |
| SO2_LAG_1 | SO2 (ppm), 1 day before the survey |
| SO2_LAG_2 | SO2 (ppm), 2 days before the survey |
| SO2_LAG_3 | SO2 (ppm), 3 days before the survey |
| SO2_LAG_4 | SO2 (ppm), 4 days before the survey |
| SO2_LAG_5 | SO2 (ppm), 5 days before the survey |
| SO2_LAG_6 | SO2 (ppm), 6 days before the survey |
| SO2_LAG_7 | SO2 (ppm), 7 days before the survey |
| SO2_LAG_8 | SO2 (ppm), 8 days before the survey |
| SO2_LAG_9 | SO2 (ppm), 9 days before the survey |
| SO2_LAG_10 | SO2 (ppm), 10 days before the survey |
| SO2_LAG_11 | SO2 (ppm), 11 days before the survey |
| SO2_LAG_12 | SO2 (ppm), 12 days before the survey |
| SO2_LAG_13 | SO2 (ppm), 13 days before the survey |
| SO2_LAG_14 | SO2 (ppm), 14 days before the survey |
| SO2_AVG_W1 | SO2 (ppm), moving average of 1 week (Lag0~Lag6) |
| SO2_AVG_M1 | SO2 (ppm), moving average of 1 month (Lag0~Lag29) |
| SO2_AVG_M3 | SO2 (ppm), moving average of 3 months (Lag0~Lag89) |
| SO2_AVG_M6 | SO2 (ppm), moving average of 6 months (Lag0~Lag179) |
| SO2_AVG_Y1 | SO2 (ppm), moving average of 1 year (Lag0~Lag364) |
| SO2_AVG_Y2 | SO2 (ppm), moving average of 2 years (Lag0~Lag729) |
| SO2_AVG_Y3 | SO2 (ppm), moving average of 3 years (Lag0~Lag1094) |
| SO2_AVG_Y2005 | SO2 (ppm), year mean of 2005 |
| SO2_AVG_Y2006 | SO2 (ppm), year mean of 2006 |
| SO2_AVG_Y2007 | SO2 (ppm), year mean of 2007 |
| SO2_AVG_Y2008 | SO2 (ppm), year mean of 2008 |
| SO2_AVG_Y2009 | SO2 (ppm), year mean of 2009 |
| SO2_AVG_Y2010 | SO2 (ppm), year mean of 2010 |
| SO2_AVG_Y2011 | SO2 (ppm), year mean of 2011 |
| SO2_AVG_Y2012 | SO2 (ppm), year mean of 2012 |
| SO2_AVG_Y2013 | SO2 (ppm), year mean of 2013 |
| SO2_AVG_Y2014 | SO2 (ppm), year mean of 2014 |
| SO2_AVG_Y2015 | SO2 (ppm), year mean of 2015 |
| SO2_AVG_Y2016 | SO2 (ppm), year mean of 2016 |
| SO2_AVG_Y2017 | SO2 (ppm), year mean of 2017 |
| NO2_LAG_0 | NO2 (ppm), the day of the survey |
| NO2_LAG_1 | NO2 (ppm), 1 day before the survey |
| NO2_LAG_2 | NO2 (ppm), 2 days before the survey |
| NO2_LAG_3 | NO2 (ppm), 3 days before the survey |
| NO2_LAG_4 | NO2 (ppm), 4 days before the survey |
| NO2_LAG_5 | NO2 (ppm), 5 days before the survey |
| NO2_LAG_6 | NO2 (ppm), 6 days before the survey |
| NO2_LAG_7 | NO2 (ppm), 7 days before the survey |
| NO2_LAG_8 | NO2 (ppm), 8 days before the survey |
| NO2_LAG_9 | NO2 (ppm), 9 days before the survey |
| NO2_LAG_10 | NO2 (ppm), 10 days before the survey |
| NO2_LAG_11 | NO2 (ppm), 11 days before the survey |
| NO2_LAG_12 | NO2 (ppm), 12 days before the survey |
| NO2_LAG_13 | NO2 (ppm), 13 days before the survey |
| NO2_LAG_14 | NO2 (ppm), 14 days before the survey |
| NO2_AVG_W1 | NO2 (ppm), moving average of 1 week (Lag0~Lag6) |
| NO2_AVG_M1 | NO2 (ppm), moving average of 1 month (Lag0~Lag29) |
| NO2_AVG_M3 | NO2 (ppm), moving average of 3 months (Lag0~Lag89) |
| NO2_AVG_M6 | NO2 (ppm), moving average of 6 months (Lag0~Lag179) |
| NO2_AVG_Y1 | NO2 (ppm), moving average of 1 year (Lag0~Lag364) |
| NO2_AVG_Y2 | NO2 (ppm), moving average of 2 years (Lag0~Lag729) |
| NO2_AVG_Y3 | NO2 (ppm), moving average of 3 years (Lag0~Lag1094) |
| NO2_AVG_Y2005 | NO2 (ppm), year mean of 2005 |
| NO2_AVG_Y2006 | NO2 (ppm), year mean of 2006 |
| NO2_AVG_Y2007 | NO2 (ppm), year mean of 2007 |
| NO2_AVG_Y2008 | NO2 (ppm), year mean of 2008 |
| NO2_AVG_Y2009 | NO2 (ppm), year mean of 2009 |
| NO2_AVG_Y2010 | NO2 (ppm), year mean of 2010 |
| NO2_AVG_Y2011 | NO2 (ppm), year mean of 2011 |
| NO2_AVG_Y2012 | NO2 (ppm), year mean of 2012 |
| NO2_AVG_Y2013 | NO2 (ppm), year mean of 2013 |
| NO2_AVG_Y2014 | NO2 (ppm), year mean of 2014 |
| NO2_AVG_Y2015 | NO2 (ppm), year mean of 2015 |
| NO2_AVG_Y2016 | NO2 (ppm), year mean of 2016 |
| NO2_AVG_Y2017 | NO2 (ppm), year mean of 2017 |
| CO_LAG_0 | CO (ppm), the day of the survey |
| CO_LAG_1 | CO (ppm), 1 day before the survey |
| CO_LAG_2 | CO (ppm), 2 days before the survey |
| CO_LAG_3 | CO (ppm), 3 days before the survey |
| CO_LAG_4 | CO (ppm), 4 days before the survey |
| CO_LAG_5 | CO (ppm), 5 days before the survey |
| CO_LAG_6 | CO (ppm), 6 days before the survey |
| CO_LAG_7 | CO (ppm), 7 days before the survey |
| CO_LAG_8 | CO (ppm), 8 days before the survey |
| CO_LAG_9 | CO (ppm), 9 days before the survey |
| CO_LAG_10 | CO (ppm), 10 days before the survey |
| CO_LAG_11 | CO (ppm), 11 days before the survey |
| CO_LAG_12 | CO (ppm), 12 days before the survey |
| CO_LAG_13 | CO (ppm), 13 days before the survey |
| CO_LAG_14 | CO (ppm), 14 days before the survey |
| CO_AVG_W1 | CO (ppm), moving average of 1 week (Lag0~Lag6) |
| CO_AVG_M1 | CO (ppm), moving average of 1 month (Lag0~Lag29) |
| CO_AVG_M3 | CO (ppm), moving average of 3 months (Lag0~Lag89) |
| CO_AVG_M6 | CO (ppm), moving average of 6 months (Lag0~Lag179) |
| CO_AVG_Y1 | CO (ppm), moving average of 1 year (Lag0~Lag364) |
| CO_AVG_Y2 | CO (ppm), moving average of 2 years (Lag0~Lag729) |
| CO_AVG_Y3 | CO (ppm), moving average of 3 years (Lag0~Lag1094) |
| CO_AVG_Y2005 | CO (ppm), year mean of 2005 |
| CO_AVG_Y2006 | CO (ppm), year mean of 2006 |
| CO_AVG_Y2007 | CO (ppm), year mean of 2007 |
| CO_AVG_Y2008 | CO (ppm), year mean of 2008 |
| CO_AVG_Y2009 | CO (ppm), year mean of 2009 |
| CO_AVG_Y2010 | CO (ppm), year mean of 2010 |
| CO_AVG_Y2011 | CO (ppm), year mean of 2011 |
| CO_AVG_Y2012 | CO (ppm), year mean of 2012 |
| CO_AVG_Y2013 | CO (ppm), year mean of 2013 |
| CO_AVG_Y2014 | CO (ppm), year mean of 2014 |
| CO_AVG_Y2015 | CO (ppm), year mean of 2015 |
| CO_AVG_Y2016 | CO (ppm), year mean of 2016 |
| CO_AVG_Y2017 | CO (ppm), year mean of 2017 |
| O3_LAG_0 | O3 (ppm), the day of the survey |
| O3_LAG_1 | O3 (ppm), 1 day before the survey |
| O3_LAG_2 | O3 (ppm), 2 days before the survey |
| O3_LAG_3 | O3 (ppm), 3 days before the survey |
| O3_LAG_4 | O3 (ppm), 4 days before the survey |
| O3_LAG_5 | O3 (ppm), 5 days before the survey |
| O3_LAG_6 | O3 (ppm), 6 days before the survey |
| O3_LAG_7 | O3 (ppm), 7 days before the survey |
| O3_LAG_8 | O3 (ppm), 8 days before the survey |
| O3_LAG_9 | O3 (ppm), 9 days before the survey |
| O3_LAG_10 | O3 (ppm), 10 days before the survey |
| O3_LAG_11 | O3 (ppm), 11 days before the survey |
| O3_LAG_12 | O3 (ppm), 12 days before the survey |
| O3_LAG_13 | O3 (ppm), 13 days before the survey |
| O3_LAG_14 | O3 (ppm), 14 days before the survey |
| O3_AVG_W1 | O3 (ppm), moving average of 1 week (Lag0~Lag6) |
| O3_AVG_M1 | O3 (ppm), moving average of 1 month (Lag0~Lag29) |
| O3_AVG_M3 | O3 (ppm), moving average of 3 months (Lag0~Lag89) |
| O3_AVG_M6 | O3 (ppm), moving average of 6 months (Lag0~Lag179) |
| O3_AVG_Y1 | O3 (ppm), moving average of 1 year (Lag0~Lag364) |
| O3_AVG_Y2 | O3 (ppm), moving average of 2 years (Lag0~Lag729) |
| O3_AVG_Y3 | O3 (ppm), moving average of 3 years (Lag0~Lag1094) |
| O3_AVG_Y2005 | O3 (ppm), year mean of 2005 |
| O3_AVG_Y2006 | O3 (ppm), year mean of 2006 |
| O3_AVG_Y2007 | O3 (ppm), year mean of 2007 |
| O3_AVG_Y2008 | O3 (ppm), year mean of 2008 |
| O3_AVG_Y2009 | O3 (ppm), year mean of 2009 |
| O3_AVG_Y2010 | O3 (ppm), year mean of 2010 |
| O3_AVG_Y2011 | O3 (ppm), year mean of 2011 |
| O3_AVG_Y2012 | O3 (ppm), year mean of 2012 |
| O3_AVG_Y2013 | O3 (ppm), year mean of 2013 |
| O3_AVG_Y2014 | O3 (ppm), year mean of 2014 |
| O3_AVG_Y2015 | O3 (ppm), year mean of 2015 |
| O3_AVG_Y2016 | O3 (ppm), year mean of 2016 |
| O3_AVG_Y2017 | O3 (ppm), year mean of 2017 |
| *Meteorological variables* |  |
| TEMP_LAG_0 | Temperature (℃), the day of the survey |
| TEMPMIN_LAG_0 | Lowest temperature (℃), the day of the survey |
| TEMPMAX_LAG_0 | Highest temperature (℃), the day of the survey |
| TEMP_LAG_1 | Temperature (℃), 1 day before the survey |
| TEMPMIN_LAG_1 | Lowest temperature (℃), 1 day before the survey |
| TEMPMAX_LAG_1 | Highest temperature (℃), 1 day before the survey |
| TEMP_LAG_2 | Temperature (℃), 2 days before the survey |
| TEMPMIN_LAG_2 | Lowest temperature (℃), 2 days before the survey |
| TEMPMAX_LAG_2 | Highest temperature (℃), 2 days before the survey |
| TEMP_LAG_3 | Temperature (℃), 3 days before the survey |
| TEMPMIN_LAG_3 | Lowest temperature (℃), 3 days before the survey |
| TEMPMAX_LAG_3 | Highest temperature (℃), 3 days before the survey |
| TEMP_LAG_4 | Temperature (℃), 4 days before the survey |
| TEMPMIN_LAG_4 | Lowest temperature (℃), 4 days before the survey |
| TEMPMAX_LAG_4 | Highest temperature (℃), 4 days before the survey |
| TEMP_LAG_5 | Temperature (℃), 5 days before the survey |
| TEMPMIN_LAG_5 | Lowest temperature (℃), 5 days before the survey |
| TEMPMAX_LAG_5 | Highest temperature (℃), 5 days before the survey |
| TEMP_LAG_6 | Temperature (℃), 6 days before the survey |
| TEMPMIN_LAG_6 | Lowest temperature (℃), 6 days before the survey |
| TEMPMAX_LAG_6 | Highest temperature (℃), 6 days before the survey |
| TEMP_LAG_7 | Temperature (℃), 7 days before the survey |
| TEMPMIN_LAG_7 | Lowest temperature (℃), 7 days before the survey |
| TEMPMAX_LAG_7 | Highest temperature (℃), 7 days before the survey |
| TEMP_LAG_8 | Temperature (℃), 8 days before the survey |
| TEMPMIN_LAG_8 | Lowest temperature (℃), 8 days before the survey |
| TEMPMAX_LAG_8 | Highest temperature (℃), 8 days before the survey |
| TEMP_LAG_9 | Temperature (℃), 9 days before the survey |
| TEMPMIN_LAG_9 | Lowest temperature (℃), 9 days before the survey |
| TEMPMAX_LAG_9 | Highest temperature (℃), 9 days before the survey |
| TEMP_LAG_10 | Temperature (℃), 10 days before the survey |
| TEMPMIN_LAG_10 | Lowest temperature (℃), 10 days before the survey |
| TEMPMAX_LAG_10 | Highest temperature (℃), 10 days before the survey |
| TEMP_LAG_11 | Temperature (℃), 11 days before the survey |
| TEMPMIN_LAG_11 | Lowest temperature (℃), 11 days before the survey |
| TEMPMAX_LAG_11 | Highest temperature (℃), 11 days before the survey |
| TEMP_LAG_12 | Temperature (℃), 12 days before the survey |
| TEMPMIN_LAG_12 | Lowest temperature (℃), 12 days before the survey |
| TEMPMAX_LAG_12 | Highest temperature (℃), 12 days before the survey |
| TEMP_LAG_13 | Temperature (℃), 13 days before the survey |
| TEMPMIN_LAG_13 | Lowest temperature (℃), 13 days before the survey |
| TEMPMAX_LAG_13 | Highest temperature (℃), 13 days before the survey |
| TEMP_LAG_14 | Temperature (℃), 14 days before the survey |
| TEMPMIN_LAG_14 | Lowest temperature (℃), 14 days before the survey |
| TEMPMAX_LAG_14 | Highest temperature (℃), 14 days before the survey |
| TEMP_AVG_W1 | Temperature (℃), moving average of 1 week (Lag0~Lag6) |
| TEMP_AVG_M1 | Temperature (℃), moving average of 1 month (Lag0~Lag29) |
| TEMP_AVG_M3 | Temperature (℃), moving average of 3 months (Lag0~Lag89) |
| TEMP_AVG_M6 | Temperature (℃), moving average of 6 months (Lag0~Lag179) |
| TEMP_AVG_Y1 | Temperature (℃), moving average of 1 year (Lag0~Lag364) |
| TEMP_AVG_Y2 | Temperature (℃), moving average of 2 years (Lag0~Lag729) |
| TEMP_AVG_Y3 | Temperature (℃), moving average of 3 years (Lag0~Lag1094) |
| TEMP_AVG_Y2005 | Temperature (℃), year mean of 2005 |
| TEMP_AVG_Y2006 | Temperature (℃), year mean of 2006 |
| TEMP_AVG_Y2007 | Temperature (℃), year mean of 2007 |
| TEMP_AVG_Y2008 | Temperature (℃), year mean of 2008 |
| TEMP_AVG_Y2009 | Temperature (℃), year mean of 2009 |
| TEMP_AVG_Y2010 | Temperature (℃), year mean of 2010 |
| TEMP_AVG_Y2011 | Temperature (℃), year mean of 2011 |
| TEMP_AVG_Y2012 | Temperature (℃), year mean of 2012 |
| TEMP_AVG_Y2013 | Temperature (℃), year mean of 2013 |
| TEMP_AVG_Y2014 | Temperature (℃), year mean of 2014 |
| TEMP_AVG_Y2015 | Temperature (℃), year mean of 2015 |
| TEMP_AVG_Y2016 | Temperature (℃), year mean of 2016 |
| TEMP_AVG_Y2017 | Temperature (℃), year mean of 2017 |
| WS_LAG_0 | Wind speed (m/s), the day of the survey |
| WS_LAG_1 | Wind speed (m/s), 1 day before the survey |
| WS_LAG_2 | Wind speed (m/s), 2 days before the survey |
| WS_LAG_3 | Wind speed (m/s), 3 days before the survey |
| WS_LAG_4 | Wind speed (m/s), 4 days before the survey |
| WS_LAG_5 | Wind speed (m/s), 5 days before the survey |
| WS_LAG_6 | Wind speed (m/s), 6 days before the survey |
| WS_LAG_7 | Wind speed (m/s), 7 days before the survey |
| WS_LAG_8 | Wind speed (m/s), 8 days before the survey |
| WS_LAG_9 | Wind speed (m/s), 9 days before the survey |
| WS_LAG_10 | Wind speed (m/s), 10 days before the survey |
| WS_LAG_11 | Wind speed (m/s), 11 days before the survey |
| WS_LAG_12 | Wind speed (m/s), 12 days before the survey |
| WS_LAG_13 | Wind speed (m/s), 13 days before the survey |
| WS_LAG_14 | Wind speed (m/s), 14 days before the survey |
| WS_AVG_W1 | Wind speed (m/s), moving average of 1 week (Lag0~Lag6) |
| WS_AVG_M1 | Wind speed (m/s), moving average of 1 month (Lag0~Lag29) |
| WS_AVG_M3 | Wind speed (m/s), moving average of 3 months (Lag0~Lag89) |
| WS_AVG_M6 | Wind speed (m/s), moving average of 6 months (Lag0~Lag179) |
| WS_AVG_Y1 | Wind speed (m/s), moving average of 1 year (Lag0~Lag364) |
| WS_AVG_Y2 | Wind speed (m/s), moving average of 2 years (Lag0~Lag729) |
| WS_AVG_Y3 | Wind speed (m/s), moving average of 3 years (Lag0~Lag1094) |
| WS_AVG_Y2005 | Wind speed (m/s), year mean of 2005 |
| WS_AVG_Y2006 | Wind speed (m/s), year mean of 2006 |
| WS_AVG_Y2007 | Wind speed (m/s), year mean of 2007 |
| WS_AVG_Y2008 | Wind speed (m/s), year mean of 2008 |
| WS_AVG_Y2009 | Wind speed (m/s), year mean of 2009 |
| WS_AVG_Y2010 | Wind speed (m/s), year mean of 2010 |
| WS_AVG_Y2011 | Wind speed (m/s), year mean of 2011 |
| WS_AVG_Y2012 | Wind speed (m/s), year mean of 2012 |
| WS_AVG_Y2013 | Wind speed (m/s), year mean of 2013 |
| WS_AVG_Y2014 | Wind speed (m/s), year mean of 2014 |
| WS_AVG_Y2015 | Wind speed (m/s), year mean of 2015 |
| WS_AVG_Y2016 | Wind speed (m/s), year mean of 2016 |
| WS_AVG_Y2017 | Wind speed (m/s), year mean of 2017 |
| RH_LAG_0 | Humidity (%), the day of the survey |
| RH_LAG_1 | Humidity (%), 1 day before the survey |
| RH_LAG_2 | Humidity (%), 2 days before the survey |
| RH_LAG_3 | Humidity (%), 3 days before the survey |
| RH_LAG_4 | Humidity (%), 4 days before the survey |
| RH_LAG_5 | Humidity (%), 5 days before the survey |
| RH_LAG_6 | Humidity (%), 6 days before the survey |
| RH_LAG_7 | Humidity (%), 7 days before the survey |
| RH_LAG_8 | Humidity (%), 8 days before the survey |
| RH_LAG_9 | Humidity (%), 9 days before the survey |
| RH_LAG_10 | Humidity (%), 10 days before the survey |
| RH_LAG_11 | Humidity (%), 11 days before the survey |
| RH_LAG_12 | Humidity (%), 12 days before the survey |
| RH_LAG_13 | Humidity (%), 13 days before the survey |
| RH_LAG_14 | Humidity (%), 14 days before the survey |
| RH_AVG_W1 | Humidity (%), moving average of 1 week (Lag0~Lag6) |
| RH_AVG_M1 | Humidity (%), moving average of 1 month (Lag0~Lag29) |
| RH_AVG_M3 | Humidity (%), moving average of 3 months (Lag0~Lag89) |
| RH_AVG_M6 | Humidity (%), moving average of 6 months (Lag0~Lag179) |
| RH_AVG_Y1 | Humidity (%), moving average of 1 year (Lag0~Lag364) |
| RH_AVG_Y2 | Humidity (%), moving average of 2 years (Lag0~Lag729) |
| RH_AVG_Y3 | Humidity (%), moving average of 3 years (Lag0~Lag1094) |
| RH_AVG_Y2005 | Humidity (%), year mean of 2005 |
| RH_AVG_Y2006 | Humidity (%), year mean of 2006 |
| RH_AVG_Y2007 | Humidity (%), year mean of 2007 |
| RH_AVG_Y2008 | Humidity (%), year mean of 2008 |
| RH_AVG_Y2009 | Humidity (%), year mean of 2009 |
| RH_AVG_Y2010 | Humidity (%), year mean of 2010 |
| RH_AVG_Y2011 | Humidity (%), year mean of 2011 |
| RH_AVG_Y2012 | Humidity (%), year mean of 2012 |
| RH_AVG_Y2013 | Humidity (%), year mean of 2013 |
| RH_AVG_Y2014 | Humidity (%), year mean of 2014 |
| RH_AVG_Y2015 | Humidity (%), year mean of 2015 |
| RH_AVG_Y2016 | Humidity (%), year mean of 2016 |
| RH_AVG_Y2017 | Humidity (%), year mean of 2017 |
| RN_LAG_0 | precipitation (cm/hr), the day of the survey |
| RN_LAG_1 | precipitation (cm/hr), 1 day before the survey |
| RN_LAG_2 | precipitation (cm/hr), 2 days before the survey |
| RN_LAG_3 | precipitation (cm/hr), 3 days before the survey |
| RN_LAG_4 | precipitation (cm/hr), 4 days before the survey |
| RN_LAG_5 | precipitation (cm/hr), 5 days before the survey |
| RN_LAG_6 | precipitation (cm/hr), 6 days before the survey |
| RN_LAG_7 | precipitation (cm/hr), 7 days before the survey |
| RN_LAG_8 | precipitation (cm/hr), 8 days before the survey |
| RN_LAG_9 | precipitation (cm/hr), 9 days before the survey |
| RN_LAG_10 | precipitation (cm/hr), 10 days before the survey |
| RN_LAG_11 | precipitation (cm/hr), 11 days before the survey |
| RN_LAG_12 | precipitation (cm/hr), 12 days before the survey |
| RN_LAG_13 | precipitation (cm/hr), 13 days before the survey |
| RN_LAG_14 | precipitation (cm/hr), 14 days before the survey |
| RN_AVG_W1 | precipitation (cm/hr), moving average of 1 week (Lag0~Lag6) |
| RN_AVG_M1 | precipitation (cm/hr), moving average of 1 month (Lag0~Lag29) |
| RN_AVG_M3 | precipitation (cm/hr), moving average of 3 months (Lag0~Lag89) |
| RN_AVG_M6 | precipitation (cm/hr), moving average of 6 months (Lag0~Lag179) |
| RN_AVG_Y1 | precipitation (cm/hr), moving average of 1 year (Lag0~Lag364) |
| RN_AVG_Y2 | precipitation (cm/hr), moving average of 2 years (Lag0~Lag729) |
| RN_AVG_Y3 | precipitation (cm/hr), moving average of 3 years (Lag0~Lag1094) |
| RN_AVG_Y2005 | precipitation (cm/hr), year mean of 2005 |
| RN_AVG_Y2006 | precipitation (cm/hr), year mean of 2006 |
| RN_AVG_Y2007 | precipitation (cm/hr), year mean of 2007 |
| RN_AVG_Y2008 | precipitation (cm/hr), year mean of 2008 |
| RN_AVG_Y2009 | precipitation (cm/hr), year mean of 2009 |
| RN_AVG_Y2010 | precipitation (cm/hr), year mean of 2010 |
| RN_AVG_Y2011 | precipitation (cm/hr), year mean of 2011 |
| RN_AVG_Y2012 | precipitation (cm/hr), year mean of 2012 |
| RN_AVG_Y2013 | precipitation (cm/hr), year mean of 2013 |
| RN_AVG_Y2014 | precipitation (cm/hr), year mean of 2014 |
| RN_AVG_Y2015 | precipitation (cm/hr), year mean of 2015 |
| RN_AVG_Y2016 | precipitation (cm/hr), year mean of 2016 |
| RN_AVG_Y2017 | precipitation (cm/hr), year mean of 2017 |
| CA_LAG_0 | Cloud Fraction, the day of the survey |
| CA_LAG_1 | Cloud Fraction, 1 day before the survey |
| CA_LAG_2 | Cloud Fraction, 2 days before the survey |
| CA_LAG_3 | Cloud Fraction, 3 days before the survey |
| CA_LAG_4 | Cloud Fraction, 4 days before the survey |
| CA_LAG_5 | Cloud Fraction, 5 days before the survey |
| CA_LAG_6 | Cloud Fraction, 6 days before the survey |
| CA_LAG_7 | Cloud Fraction, 7 days before the survey |
| CA_LAG_8 | Cloud Fraction, 8 days before the survey |
| CA_LAG_9 | Cloud Fraction, 9 days before the survey |
| CA_LAG_10 | Cloud Fraction, 10 days before the survey |
| CA_LAG_11 | Cloud Fraction, 11 days before the survey |
| CA_LAG_12 | Cloud Fraction, 12 days before the survey |
| CA_LAG_13 | Cloud Fraction, 13 days before the survey |
| CA_LAG_14 | Cloud Fraction, 14 days before the survey |
| CA_AVG_W1 | Cloud Fraction, moving average of 1 week (Lag0~Lag6) |
| CA_AVG_M1 | Cloud Fraction, moving average of 1 month (Lag0~Lag29) |
| CA_AVG_M3 | Cloud Fraction, moving average of 3 months (Lag0~Lag89) |
| CA_AVG_M6 | Cloud Fraction, moving average of 6 months (Lag0~Lag179) |
| CA_AVG_Y1 | Cloud Fraction, moving average of 1 year (Lag0~Lag364) |
| CA_AVG_Y2 | Cloud Fraction, moving average of 2 years (Lag0~Lag729) |
| CA_AVG_Y3 | Cloud Fraction, moving average of 3 years (Lag0~Lag1094) |
| CA_AVG_Y2005 | Cloud Fraction, year mean of 2005 |
| CA_AVG_Y2006 | Cloud Fraction, year mean of 2006 |
| CA_AVG_Y2007 | Cloud Fraction, year mean of 2007 |
| CA_AVG_Y2008 | Cloud Fraction, year mean of 2008 |
| CA_AVG_Y2009 | Cloud Fraction, year mean of 2009 |
| CA_AVG_Y2010 | Cloud Fraction, year mean of 2010 |
| CA_AVG_Y2011 | Cloud Fraction, year mean of 2011 |
| CA_AVG_Y2012 | Cloud Fraction, year mean of 2012 |
| CA_AVG_Y2013 | Cloud Fraction, year mean of 2013 |
| CA_AVG_Y2014 | Cloud Fraction, year mean of 2014 |
| CA_AVG_Y2015 | Cloud Fraction, year mean of 2015 |
| CA_AVG_Y2016 | Cloud Fraction, year mean of 2016 |
| CA_AVG_Y2017 | Cloud Fraction, year mean of 2017 |
| WD_LAG_0 | Wind direction (degree), the day of the survey |
| WD_LAG_1 | Wind direction (degree), 1 day before the survey |
| WD_LAG_2 | Wind direction (degree), 2 days before the survey |
| WD_LAG_3 | Wind direction (degree), 3 days before the survey |
| WD_LAG_4 | Wind direction (degree), 4 days before the survey |
| WD_LAG_5 | Wind direction (degree), 5 days before the survey |
| WD_LAG_6 | Wind direction (degree), 6 days before the survey |
| WD_LAG_7 | Wind direction (degree), 7 days before the survey |
| WD_LAG_8 | Wind direction (degree), 8 days before the survey |
| WD_LAG_9 | Wind direction (degree), 9 days before the survey |
| WD_LAG_10 | Wind direction (degree), 10 days before the survey |
| WD_LAG_11 | Wind direction (degree), 11 days before the survey |
| WD_LAG_12 | Wind direction (degree), 12 days before the survey |
| WD_LAG_13 | Wind direction (degree), 13 days before the survey |
| WD_LAG_14 | Wind direction (degree), 14 days before the survey |
| GSW_LAG_0 | Insolation (W/㎡), the day of the survey |
| GSW_LAG_1 | Insolation (W/㎡), 1 day before the survey |
| GSW_LAG_2 | Insolation (W/㎡), 2 days before the survey |
| GSW_LAG_3 | Insolation (W/㎡), 3 days before the survey |
| GSW_LAG_4 | Insolation (W/㎡), 4 days before the survey |
| GSW_LAG_5 | Insolation (W/㎡), 5 days before the survey |
| GSW_LAG_6 | Insolation (W/㎡), 6 days before the survey |
| GSW_LAG_7 | Insolation (W/㎡), 7 days before the survey |
| GSW_LAG_8 | Insolation (W/㎡), 8 days before the survey |
| GSW_LAG_9 | Insolation (W/㎡), 9 days before the survey |
| GSW_LAG_10 | Insolation (W/㎡), 10 days before the survey |
| GSW_LAG_11 | Insolation (W/㎡), 11 days before the survey |
| GSW_LAG_12 | Insolation (W/㎡), 12 days before the survey |
| GSW_LAG_13 | Insolation (W/㎡), 13 days before the survey |
| GSW_LAG_14 | Insolation (W/㎡), 14 days before the survey |
| GSW_AVG_W1 | Insolation (W/㎡), moving average of 1 week (Lag0~Lag6) |
| GSW_AVG_M1 | Insolation (W/㎡), moving average of 1 month (Lag0~Lag29) |
| GSW_AVG_M3 | Insolation (W/㎡), moving average of 3 months (Lag0~Lag89) |
| GSW_AVG_M6 | Insolation (W/㎡), moving average of 6 months (Lag0~Lag179) |
| GSW_AVG_Y1 | Insolation (W/㎡), moving average of 1 year (Lag0~Lag364) |
| GSW_AVG_Y2 | Insolation (W/㎡), moving average of 2 years (Lag0~Lag729) |
| GSW_AVG_Y3 | Insolation (W/㎡), moving average of 3 years (Lag0~Lag1094) |
| GSW_AVG_Y2005 | Insolation (W/㎡), year mean of 2005 |
| GSW_AVG_Y2006 | Insolation (W/㎡), year mean of 2006 |
| GSW_AVG_Y2007 | Insolation (W/㎡), year mean of 2007 |
| GSW_AVG_Y2008 | Insolation (W/㎡), year mean of 2008 |
| GSW_AVG_Y2009 | Insolation (W/㎡), year mean of 2009 |
| GSW_AVG_Y2010 | Insolation (W/㎡), year mean of 2010 |
| GSW_AVG_Y2011 | Insolation (W/㎡), year mean of 2011 |
| GSW_AVG_Y2012 | Insolation (W/㎡), year mean of 2012 |
| GSW_AVG_Y2013 | Insolation (W/㎡), year mean of 2013 |
| GSW_AVG_Y2014 | Insolation (W/㎡), year mean of 2014 |
| GSW_AVG_Y2015 | Insolation (W/㎡), year mean of 2015 |
| GSW_AVG_Y2016 | Insolation (W/㎡), year mean of 2016 |
| GSW_AVG_Y2017 | Insolation (W/㎡), year mean of 2017 |
| PRESFC_LAG_0 | Surface pressure (hPa), the day of the survey |
| PRESFC_LAG_1 | Surface pressure (hPa), 1 day before the survey |
| PRESFC_LAG_2 | Surface pressure (hPa), 2 days before the survey |
| PRESFC_LAG_3 | Surface pressure (hPa), 3 days before the survey |
| PRESFC_LAG_4 | Surface pressure (hPa), 4 days before the survey |
| PRESFC_LAG_5 | Surface pressure (hPa), 5 days before the survey |
| PRESFC_LAG_6 | Surface pressure (hPa), 6 days before the survey |
| PRESFC_LAG_7 | Surface pressure (hPa), 7 days before the survey |
| PRESFC_LAG_8 | Surface pressure (hPa), 8 days before the survey |
| PRESFC_LAG_9 | Surface pressure (hPa), 9 days before the survey |
| PRESFC_LAG_10 | Surface pressure (hPa), 10 days before the survey |
| PRESFC_LAG_11 | Surface pressure (hPa), 11 days before the survey |
| PRESFC_LAG_12 | Surface pressure (hPa), 12 days before the survey |
| PRESFC_LAG_13 | Surface pressure (hPa), 13 days before the survey |
| PRESFC_LAG_14 | Surface pressure (hPa), 14 days before the survey |
| PRESFC_AVG_W1 | Surface pressure (hPa), moving average of 1 week (Lag0~Lag6) |
| PRESFC_AVG_M1 | Surface pressure (hPa), moving average of 1 month (Lag0~Lag29) |
| PRESFC_AVG_M3 | Surface pressure (hPa), moving average of 3 months (Lag0~Lag89) |
| PRESFC_AVG_M6 | Surface pressure (hPa), moving average of 6 months (Lag0~Lag179) |
| PRESFC_AVG_Y1 | Surface pressure (hPa), moving average of 1 year (Lag0~Lag364) |
| PRESFC_AVG_Y2 | Surface pressure (hPa), moving average of 2 years (Lag0~Lag729) |
| PRESFC_AVG_Y3 | Surface pressure (hPa), moving average of 3 years (Lag0~Lag1094) |
| PRESFC_AVG_Y2005 | Surface pressure (hPa), year mean of 2005 |
| PRESFC_AVG_Y2006 | Surface pressure (hPa), year mean of 2006 |
| PRESFC_AVG_Y2007 | Surface pressure (hPa), year mean of 2007 |
| PRESFC_AVG_Y2008 | Surface pressure (hPa), year mean of 2008 |
| PRESFC_AVG_Y2009 | Surface pressure (hPa), year mean of 2009 |
| PRESFC_AVG_Y2010 | Surface pressure (hPa), year mean of 2010 |
| PRESFC_AVG_Y2011 | Surface pressure (hPa), year mean of 2011 |
| PRESFC_AVG_Y2012 | Surface pressure (hPa), year mean of 2012 |
| PRESFC_AVG_Y2013 | Surface pressure (hPa), year mean of 2013 |
| PRESFC_AVG_Y2014 | Surface pressure (hPa), year mean of 2014 |
| PRESFC_AVG_Y2015 | Surface pressure (hPa), year mean of 2015 |
| PRESFC_AVG_Y2016 | Surface pressure (hPa), year mean of 2016 |
| PRESFC_AVG_Y2017 | Surface pressure (hPa), year mean of 2017 |
