# Supplementary material for: Integrated dataset of the Korean Genome and Epidemiology Study cohort with estimated air pollution data
Source: Epidemiol Health. 2022 Sep 7;44:e2022071. doi: 10.4178/epih.e2022071 (PMC9849844; doi:10.4178/epih.e2022071)
Supplement: Supplementary Material 3. — Mean exposure levels of PMs among cohort participants in baseline survey [file epih-44-e2022071-suppl3.docx]

Supplementary Material 3. Mean exposure levels of PMs among cohort participants in baseline survey

|  |  | KoGES Ansan and Ansung^1^ | | | KoGES CAVAS | | | KoGES HEXA | | |
| --- | --- | --- | --- | --- | --- | --- | --- | --- | --- | --- |
|  | Year | N | Mean(SD) | Min-Max | N | Mean(SD) | Min-Max | N | Mean(SD) | Min-Max |
| PM_2.5_, μg/m^3^ | 2005 | 3604 | 29.8(13.8) | 7.9-92.2 | 1286 | 27.0(12.6) | 6.2-88.8 | 15756 | 28.2(13.6) | 3.3-102.2 |
|  | 2006 | 3910 | 35.0(16.4) | 7.2-96.1 | 16802 | 26.2(14.2) | 1.6-109.6 | 20391 | 27.9(14.9) | 3.5-131.1 |
|  | 2007 |  |  |  | 3015 | 23.0(11.5) | 4.7-71.8 | 21587 | 26.4(13.4) | 2.8-84.8 |
|  | 2008 |  |  |  | 1713 | 29.3(11.9) | 5.1-76.1 | 23714 | 25.3(12.2) | 0.3-88.8 |
|  | 2009 |  |  |  | 1960 | 24.2(11.6) | 7.2-67.3 | 23264 | 25.1(12.4) | 1.9-98.0 |
|  | 2010 |  |  |  | 2398 | 22.4(10.3) | 5.0-84.6 | 23813 | 24.8(12.5) | 2.8-104.7 |
|  | 2011 |  |  |  | 934 | 21.9(7.3) | 8.5-45.9 | 18529 | 24.9(11.9) | 2.5-125.1 |
|  | 2012 |  |  |  | 93 | 30.4(13.6) | 12.3-73.4 | 7254 | 24.9(11.9) | 4.0-103.5 |
|  | 2013 |  |  |  |  |  |  | 4938 | 24.1(11.1) | 4.6-80.3 |
| PM_10_, μg/m^3^ | 2005 | 3604 | 56.9(24.4) | 17.6-169.0 | 1286 | 54.9(21.6) | 16.4-156.5 | 15756 | 56.7(25.0) | 7.4-185.2 |
|  | 2006 | 3910 | 62.1(26.6) | 15.3-153.3 | 16802 | 48.7(23.2) | 7.6-171.0 | 20391 | 51.1(23.2) | 9.9-201.5 |
|  | 2007 |  |  |  | 3015 | 44.9(20.5) | 11.6-123.4 | 21587 | 52.7(24.4) | 9.4-449.5 |
|  | 2008 |  |  |  | 1713 | 67.4(30.2) | 11.8-188.3 | 23714 | 52.9(23.8) | 10.6-235.9 |
|  | 2009 |  |  |  | 1960 | 45.2(19.1) | 13.2-138.7 | 23264 | 49.4(21.8) | 10.4-179.9 |
|  | 2010 |  |  |  | 2398 | 42.1(17.9) | 8.6-139.9 | 23813 | 48.3(21.5) | 8.2-263.0 |
|  | 2011 |  |  |  | 934 | 42.1(13.1) | 17.7-86.7 | 18529 | 48.3(25.5) | 10.8-362.3 |
|  | 2012 |  |  |  | 93 | 53.6(19.0) | 26.0-111.0 | 7254 | 44.3(17.7) | 10.7-150.9 |
|  | 2013 |  |  |  |  |  |  | 4938 | 45.9(18.9) | 13.2-174.7 |

^1^The 2^nd^ follow-up is considered the baseline for the KoGES Ansan-Ansung study. Mean exposure levels were calculated by year using the date of survey (lag0) in baseline. CAVAS: Cardiovascular Disease Association Study; HEXA, Health Examinee Study
