# Supplementary material for: Integrated dataset of the Korean Genome and Epidemiology Study cohort with estimated air pollution data
Source: Epidemiol Health. 2022 Sep 7;44:e2022071. doi: 10.4178/epih.e2022071 (PMC9849844; doi:10.4178/epih.e2022071)
Supplement: Supplementary Material 4. — Mean exposure levels of gaseous air pollutants among cohort participants in baseline survey [file epih-44-e2022071-suppl4.docx]

Supplementary Material 4. Mean exposure levels of gaseous air pollutants among cohort participants in baseline survey

|  |  | KoGES Ansan and Ansung^1^ | | | KoGES CAVAS | | | KoGES HEXA | | |
| --- | --- | --- | --- | --- | --- | --- | --- | --- | --- | --- |
|  | Year | N | Mean(SD) | Min-Max | N | Mean(SD) | Min-Max | N | Mean(SD) | Min-Max |
| NO_2_, ppm | 2005 | 3604 | 0.022(0.011) | 0.0055-0.0669 | 1286 | 0.020(0.010) | 0.0039-0.0526 | 15756 | 0.025(0.013) | 0.0002-0.0758 |
|  | 2006 | 3910 | 0.024(0.010) | 0.0028-0.0629 | 16802 | 0.016(0.009) | 0.0001-0.0693 | 20391 | 0.026(0.013) | 0.0002-0.0747 |
|  | 2007 |  |  |  | 3015 | 0.014(0.010) | 0.0011-0.0501 | 21587 | 0.026(0.013) | 0.0001-0.1141 |
|  | 2008 |  |  |  | 1713 | 0.025(0.012) | 0.0003-0.0629 | 23714 | 0.026(0.013) | 0.0002-0.0798 |
|  | 2009 |  |  |  | 1960 | 0.012(0.005) | 0.0023-0.0307 | 23264 | 0.024(0.013) | 0.0001-0.0910 |
|  | 2010 |  |  |  | 2398 | 0.011(0.006) | 0.0010-0.0453 | 23813 | 0.024(0.013) | 0.0001-0.0824 |
|  | 2011 |  |  |  | 934 | 0.013(0.005) | 0.0025-0.0334 | 18529 | 0.023(0.013) | 0.0002-0.0772 |
|  | 2012 |  |  |  | 93 | 0.014(0.007) | 0.0044-0.0287 | 7254 | 0.023(0.012) | 0.0003-0.0833 |
|  | 2013 |  |  |  |  |  |  | 4938 | 0.022(0.012) | 0.0014-0.0939 |
| SO_2_, ppm | 2005 | 3604 | 0.005(0.002) | 0.0010-0.0221 | 1286 | 0.006(0.003) | 0.0014-0.0174 | 15756 | 0.006(0.004) | 0.0002-0.0438 |
|  | 2006 | 3910 | 0.005(0.002) | 0.0011-0.0143 | 16802 | 0.005(0.003) | 0.0000-0.0277 | 20391 | 0.005(0.003) | 0.0001-0.0367 |
|  | 2007 |  |  |  | 3015 | 0.004(0.003) | 0.0001-0.0171 | 21587 | 0.005(0.003) | 0.0000-0.0327 |
|  | 2008 |  |  |  | 1713 | 0.008(0.005) | 0.0003-0.0229 | 23714 | 0.005(0.003) | 0.0000-0.0393 |
|  | 2009 |  |  |  | 1960 | 0.003(0.001) | 0.0008-0.0076 | 23264 | 0.005(0.003) | 0.0001-0.0771 |
|  | 2010 |  |  |  | 2398 | 0.003(0.002) | 0.0005-0.0178 | 23813 | 0.005(0.003) | 0.0000-0.0339 |
|  | 2011 |  |  |  | 934 | 0.003(0.001) | 0.0002-0.0100 | 18529 | 0.005(0.003) | 0.0001-0.1288 |
|  | 2012 |  |  |  | 93 | 0.007(0.004) | 0.0029-0.0212 | 7254 | 0.005(0.003) | 0.0001-0.0292 |
|  | 2013 |  |  |  |  |  |  | 4938 | 0.006(0.003) | 0.0006-0.0208 |
| O_3_, ppm | 2005 | 3604 | 0.022(0.008) | 0.0054-0.0430 | 1286 | 0.019(0.005) | 0.0063-0.0265 | 15756 | 0.016(0.006) | 0.0035-0.0484 |
|  | 2006 | 3910 | 0.022(0.009) | 0.0033-0.0441 | 16802 | 0.022(0.010) | 0.0026-0.0592 | 20391 | 0.019(0.009) | 0.0019-0.0740 |
|  | 2007 |  |  |  | 3015 | 0.020(0.006) | 0.0037-0.0372 | 21587 | 0.023(0.009) | 0.0038-0.0561 |
|  | 2008 |  |  |  | 1713 | 0.019(0.008) | 0.0060-0.0439 | 23714 | 0.023(0.009) | 0.0038-0.0465 |
|  | 2009 |  |  |  | 1960 | 0.024(0.010) | 0.0047-0.0603 | 23264 | 0.024(0.012) | 0.0017-0.0991 |
|  | 2010 |  |  |  | 2398 | 0.021(0.009) | 0.0019-0.0577 | 23813 | 0.023(0.009) | 0.0024-0.0591 |
|  | 2011 |  |  |  | 934 | 0.025(0.007) | 0.0119-0.0395 | 18529 | 0.024(0.009) | 0.0040-0.0760 |
|  | 2012 |  |  |  | 93 | 0.023(0.005) | 0.0135-0.0307 | 7254 | 0.026(0.010) | 0.0047-0.0653 |
|  | 2013 |  |  |  |  |  |  | 4938 | 0.027(0.012) | 0.0036-0.0748 |
| CO, ppm | 2005 | 3604 | 0.500(0.207) | 0.215-1.654 | 1286 | 0.754(0.327) | 0.293-1.549 | 15756 | 0.555(0.283) | 0.086-1.751 |
|  | 2006 | 3910 | 0.501(0.167) | 0.182-1.502 | 16802 | 0.562(0.306) | 0.073-2.998 | 20391 | 0.542(0.238) | 0.068-2.597 |
|  | 2007 |  |  |  | 3015 | 0.528(0.324) | 0.089-2.084 | 21587 | 0.488(0.212) | 0.074-1.760 |
|  | 2008 |  |  |  | 1713 | 0.814(0.420) | 0.112-2.538 | 23714 | 0.500(0.216) | 0.080-1.852 |
|  | 2009 |  |  |  | 1960 | 0.479(0.256) | 0.154-1.412 | 23264 | 0.495(0.236) | 0.078-1.913 |
|  | 2010 |  |  |  | 2398 | 0.369(0.202) | 0.105-1.874 | 23813 | 0.447(0.205) | 0.074-2.340 |
|  | 2011 |  |  |  | 934 | 0.334(0.125) | 0.084-0.933 | 18529 | 0.421(0.170) | 0.074-1.877 |
|  | 2012 |  |  |  | 93 | 0.621(0.194) | 0.376-1.086 | 7254 | 0.463(0.189) | 0.084-1.851 |
|  | 2013 |  |  |  |  |  |  | 4938 | 0.477(0.176) | 0.121-1.424 |

^1^The 2^nd^ follow-up is considered the baseline for the KoGES Ansan-Ansung study. Mean exposure levels were calculated by year using the date of survey (lag0) in baseline. CAVAS: Cardiovascular Disease Association Study; HEXA, Health Examinee Study
