# Supplementary material for: Integrated dataset of the Korean Genome and Epidemiology Study cohort with estimated air pollution data
Source: Epidemiol Health. 2022 Sep 7;44:e2022071. doi: 10.4178/epih.e2022071 (PMC9849844; doi:10.4178/epih.e2022071)
Supplement: Supplementary Material 6. — PM10 exposure levels of the KoGES baseline participants by season and year. KoGES Ansan and Ansung study (2nd follow-up, gray), KoGES CAVAS (green), KoGES HEXA (blue). Sp: Spring, S: Summer, F: Fall, W: Winter [file epih-44-e2022071-suppl6.pptx]

## Slide 1
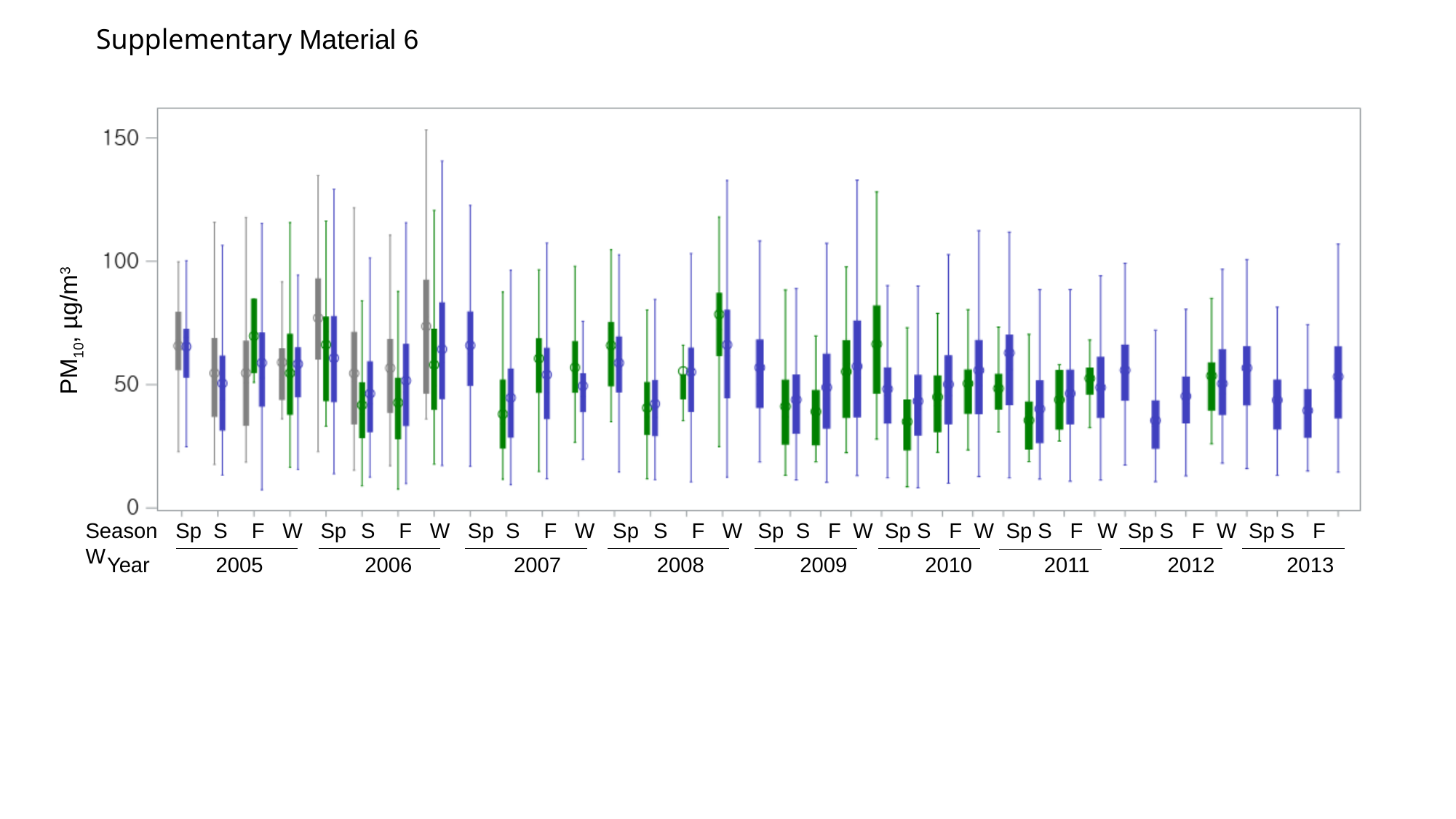

Supplementary Material 6
PM10, µg/m3
Season Sp S F W Sp S F W Sp S F W Sp S F W Sp S F W Sp S F W Sp S F W Sp S F W Sp S F W
 Year 2005 2006 2007 2008 2009 2010 2011 2012 2013
